# Factors associated with severe forms and deaths from schistosomiasis and application of probabilistic linkage in databases, state of Pernambuco, Brazil, 2007–2017

**DOI:** 10.1590/1980-549720230003.2

**Published:** 2023-01-09

**Authors:** Maria Isabelle Barbosa da Silva Brito, Emília Carolle Azevedo de Oliveira, Constança Simões Barbosa, Elainne Christine de Souza Gomes

**Affiliations:** I Fundação Oswaldo Cruz, Instituto Aggeu Magalhães – Recife (PE), Brasil.

**Keywords:** Schistosomiasis, Mortality, Health information systems, Temporal distribution, Esquistossomose, Mortalidade, Sistemas de informação em saúde, Distribuição temporal

## Abstract

**Objective:**

To verify the agreement of data on severe forms and deaths from schistosomiasis recorded in the Brazilian Notifiable Diseases Information System and the Mortality Information System, sociodemographic variables with the occurrence of severe forms and deaths, and the temporal trend of the disease in the state of Pernambuco, Brazil.

**Methods:**

This is an ecological, descriptive, time series study with data on severe forms and deaths from schistosomiasis in Pernambuco, from 2007 to 2017. For the *linkage* between databases, a function was developed in *python* programming language, using the *Soundex* method. To identify sociodemographic and health factors that correlated with the dependent variables, Pearson’s correlation test was applied. For trend analysis, linear regression was applied.

**Results:**

We identified 9,085 severe cases, 1,956 deaths, and 186 cases in the *linkage*. The correlation between the average positivity rate with the general water supply and waste collection was 0.22 and 0.26 respectively. We verified a correlation of the average cumulative mortality rate with water supply by well or spring (r=0.27), water supply by the general network (r=0.3), waste collection (r=0.42), and road urbanization (r=0.29). We found 3,153 severe forms in 2007 with a decrease trend and 205 deaths in 2010, without a trend pattern.

**Conclusion:**

There is a need for greater investments in disease control and in the quality of information, especially in the record of severe forms, considering that, due to the pathophysiology of the disease, death only occurs when the individual develops the chronic form, and its notification on the Notifiable Diseases Information System is imperative.

## RESUMO

**Objetivo:**

Verificar a concordância dos dados relacionados às formas graves e aos óbitos por esquistossomose registrados no Sistema de Informação de Agravos de Notificação e no Sistema de Informação Sobre Mortalidade, as variáveis sociodemográficas com a ocorrência das formas graves e óbitos e a tendência temporal da doença em Pernambuco.

**Métodos:**

Trata-se de um estudo ecológico, descritivo, de série temporal com dados referentes às formas graves e aos óbitos por esquistossomose em Pernambuco, de 2007 a 2017. Para o *linkage* entre as bases de dados, foi desenvolvida uma função em linguagem *python* de programação, utilizando o método *soundex*. Para identificar fatores sociodemográficos e sanitários que se correlacionaram com as variáveis dependentes, foi aplicado o teste de correlação de Pearson. Para a análise de tendência, aplicou-se a regressão linear.

**Resultados:**

Identificaram-se 9.085 casos graves, 1.956 óbitos e 186 casos no *linkage*. A correlação entre a taxa média de positividade com o abastecimento hídrico rede geral e coleta de lixo foi 0,22 e 0,26 respectivamente. Houve correlação da taxa média de mortalidade acumulada com abastecimento hídrico por poço ou nascente (r=0,27), abastecimento hídrico por rede geral (r=0,3), coleta de lixo (r=0,42), urbanização das vias (r=0,29). Ocorreram 3.153 formas graves em 2007, com tendência a redução, e 205 óbitos em 2010, sem um padrão de tendência.

**Conclusão:**

Há necessidade de maiores investimentos no controle da doença e na qualidade da informação, especialmente no registro das formas graves, pois, dada a fisiopatologia da doença, o óbito só ocorre quando o indivíduo desenvolve a forma crônica, sendo imperativo sua notificação no Sistema de Informação de Agravos de Notificação.

## INTRODUCTION

Schistosomiasis is a parasitic disease of chronic evolution, caused by digenetic trematode parasites of the genus *Schistosoma mansoni*, the only species present in the American continent^1,2^.

This disease presents different clinical forms, ranging from asymptomatic to severe forms; its severity depends on the clinical period in which it is located, and it can be classified into two stages: initial or acute and late or chronic^3^. In its chronic form, schistosomiasis manifests in human beings under two different characterizations, the hepatointestinal and hepatosplenic (compensated or decompensated), the latter is associated with high levels of parasitic load and is considered as more severe, as it is responsible for most deaths. It is characterized by massive splenomegaly, enlarged liver (which may also be contracted by perivascular fibrosis), collateral circulation, hematemesis, esophageal varices, ascites, jaundice, malnutrition, severe anemia, and hypersplenism^1-3^.

Mostly, the occurrence of schistosomiasis cases is related to leisure or work activities, as well as to practices that allow contact with contaminated waters, especially where domestic sewage is discharged^4,5^. According to the World Health Organization (WHO)^6^, this parasitosis affects approximately 240 million people worldwide, most cases are caused by *S. mansoni*, and over 700 million live in endemic territories at risk of contracting the infection. These are places with great social inequalities and weaknesses in basic sanitation, which classifies the disease as an important public health issue in several parts of the world^6^.

In Brazil, with the actions of the Schistosomiasis Control Program (*Programa de Controle da Esquistossomose*– PCE), implemented in the 1980s, the severity rates have decreased. In addition, the advance in knowledge of the natural history of the disease and the expansion of health services, with diagnosis and treatment, led to a reduction in mortality and increased survival of infected individuals^7^. However, the transmission of the disease is observed in 19 of the 27 Brazilian states, being considered the country most affected by schistosomiasis in in the Americas, with 42.9 million people at risk of infection and approximately 1.5 million infected individuals^8-10^.

According to the National Survey of Prevalence of Schistosomiasis and Geohelminths (*Inquérito Nacional de Prevalência da Esquistossomose Mansoni e Geohelmintoses*), the states with the highest percentages of positive cases were: Sergipe (8.19%), Minas Gerais (3.86%), Alagoas (2.31%), Bahia (2.19%), and Pernambuco (2.14%)^9^. The average prevalence of schistosomiasis by state diminishes the importance of epidemiological information, masking the situation of hyperendemic localities. For instance, we can mention the case of Pernambuco, which presented an average prevalence of 2% in the aforementioned survey; however, a study conducted by the Program to Combat Neglected Diseases (*Programa de enfrentamento às doenças negligenciadas*) in 2014 identified 56 locations distributed in 26 municipalities with positivity above 10%, which indicates the persistence of the severity of the disease in localized conditions^11^.

The Northeast region comprises 72% of the total cases of schistosomiasis, occurring in all its states, six of which (Alagoas, Bahia, Paraíba, Pernambuco, Rio Grande do Norte, and Sergipe) are classified as endemic; and three (Ceará, Maranhão, and Piauí) are deemed as having focal transmission of the disease^8,12^.

From 1999 to 2014, 1,943 cases of schistosomiasis were treated at Hospital das Clínicas de Pernambuco, 1,411 of which were classified as chronic^13^. Between 2013 and 2017, Pernambuco had the highest mortality rate when compared with the other rates in the Northeast and the country^14^.

Thus, information on severe forms and mortality from schistosomes evidences a neglected disease, being indispensable the monitoring of the dynamics of its occurrence as well as the review of public policy actions aimed at reducing them^1,15,16^. In this context, health information systems (HIS) emerge as active tools for the diagnosis and intervention of the health situation of the community^17^.

In the analysis of the HIS database, such as the Brazilian Notifiable Diseases Information System (*Sistema de Informação de Agravos de Notificação* – SINAN), we expect to identify the record of severe forms of schistosomiasis or cases detected in non-endemic municipalities. In the Brazilian Mortality Information System (*Sistema de Informação Sobre Mortalidade* – SIM), there are data on deaths from the disease. These systems have been widely used in recent decades, as they allow to outline indicators to support the planning of health actions aimed at controlling aggravations in several spheres of the Government^11,18^.

Taking this into consideration, in this study we aimed to verify the agreement of data on severe forms and deaths from schistosomiasis recorded in SINAN and SIM, sociodemographic variables with the occurrence of severe forms and deaths, and the temporal trend of the disease in the state of Pernambuco.

## METHODS

This is a descriptive, ecological^19^, time series (January to December 2017) study, whose unit of analysis were the municipalities by development region in the state of Pernambuco.

Located in the Northeast region of Brazil, the state of Pernambuco borders the North, with the states of Paraíba (PB) and Ceará (CE); the East, with the Atlantic Ocean; the West, with the state of Piauí (PI); and the South, with the states of Alagoas (AL) and Bahia (BA). It has 184 municipalities and one state territory, the Fernando de Noronha archipelago. As a regionalization strategy, Pernambuco is divided into 12 development regions^20,21^.

The study population was composed of confirmed notifications of severe clinical forms and deaths from schistosomiasis in the state of Pernambuco, with records in SINAN and SIM, respectively, according to the International Classification of Diseases (ICD 10) — B65 to B65.9, in the period from 2007 to 2017. Information from the notification forms and death certificates were obtained from the Epidemiological Surveillance Coordination of the State Health Department of Pernambuco (SES-PE).

This study was carried out in three stages. In the first, to identify which deaths had been previously reported as severe forms, SIM information was cross-referenced with SINAN information. These databases do not have a unique identifier, such as the number of the Individual Taxpayer Registration (*Cadastro de Pessoas Físicas* – CPF), in such a way that the technique proposed by Camargo Júnior and Coeli^22^, using blocking and pairing processes, was employed. To this end, a function was developed in python programming language, using the Integrated Development Environment (IDE) in Visual Studio Code and Google Colaboratory, by employing the Soundex method^23,24^ to people’s names, applying 20 bytes, in such a way that it includes the maximum number of characters. The remaining was completed with zeros, not leading to problems for pairing.

There were some limitations in the databases, such as in the field of the date of birth, in which there were blank fields or without the possibility of differentiating the day from the month. Thus, only the year of birth was used. The geocode of the municipality, the individual’s sex, and the mother’s name completed the columns of the pairing.

Subsequently, a combination of files was performed to generate a new database based on the linkage file, containing the records found as true pairs. After adjustments, to exclude duplicates and inconsistencies, the new database was called “Linkage between databases.” Also at this stage, the dependent variables were estimated, and the average positivity rate of the municipalities was the ratio between the number of severe forms for the period and the estimated population of 2012^20^ (average of the period) multiplied by 11 (number of years of the series), considering 100 thousand as the multiplication factor, as follows:

$$\text{Average Positivity Rate =}\frac{\text{N° of cases of severe forms for the period}\times \text{100,000}}{\text{Estimated population 2012}\times \text{11}}$$

The average cumulative mortality rate of the municipalities was calculated by the ratio between the number of deaths for the period and the estimated population of 2012^20^ (average of the period) multiplied by 11 (number of years of the series), considering 100 thousand as the multiplication factor, as described next:

$$\text{Average Cumulative Mortality Rate =}\frac{\text{N of deaths for the period}\times \text{100,000}}{\text{Estimated population 2012}\times \text{11}}$$

Only the notification forms and death certificates of individuals with a municipality of origin in the state of Pernambuco were included. The duplicate records identified in SINAN and SIM were excluded as well as the cases whose municipality of origin was ignored and records that were not concordant after the probabilistic linkage.

For the second stage, the independent variables (sociodemographic and sanitation), listed next, were obtained based on the last census conducted by the Brazilian Institute of Geography and Statistics (IBGE)^25^:

- Human Development Index (HDI);
- Water supply by general network;
- Water supply by well or spring;
- Waste collection;
- Sanitary sewer.

To identify which sociodemographic and sanitation factors correlate with the dependent variables of the study (average positivity rate and average cumulative mortality rate due to schistosomiasis), Pearson’s correlation test, with 5% significance, obtained for the study period, was applied using the R software, by the IDE Rstudio. Pearson’s correlation coefficient (r) ranges from -1 to 1, and the closer to 0, the lower the correlation between two variables.

Thus, a Pearson’s correlation matrix was created, in which the unmeasured blank squares represented the variables that were not significant. The colored squares indicated the significant variables, in such a way that shades of blue represent negative correlations and orange shades, the positive correlations.

In the third and last stage, to determine the temporal trend, the occurrence of severe forms and deaths from schistosomiasis in Pernambuco was considered for each year from 2007 to 2017, using the linear regression model, according to which:

y(occurrenceofsevereformsofschistosomiasis)= α+ βx year

y(occurrenceofdeathsfromschistosomiasis)= α​+ βx year

being observed the value of the coefficient of determination, also called R^2^, and the analysis of residues. R^2^is defined by the percentage of response variation between 0 and 100%, in such a way that the closer to 100%, the better the model’s explanation for data variability.

The Shapiro-Wilk test with 5% significance was applied to verify whether the data were normally distributed and the adequacy of the Mann-Kendall nonparametric test. Data were considered as non-normal, and the Mann-Kendall test was subsequently applied to all study variables, in which the alternative hypothesis indicates that there is a historical trend in the data.

All calculations and graphs were made using the R software version 4.1.2.

The study was conducted according to Resolution No. 510/16 of the National Health Council of the Brazilian Ministry of Health^26^ and approved by the Research Ethics Committee of the Aggeu Magalhães Institute, under Opinion No. 4.153.667 and Certificate of Presentation for Ethical Consideration (CAAE): 32992420.2.0000.5190.

## RESULTS

From 2007 to 2017, we identified 9,085 notifications for severe forms of schistosomiasis in 140 municipalities in the state of Pernambuco, 101 of which were considered endemic and belonging to the following regions: metropolitan, Zona da Mata (coastal strip of northeastern Brazil), and part of the Agreste (narrow zone between the states of Paraíba, Pernambuco, Alagoas, Sergipe, and Bahia) (Supplementary Table 1).

We identified 1,956 deaths from schistosomiasis, recorded in SIM, distributed in 123 municipalities of Pernambuco, belonging to the Agreste, Zona da Mata, and metropolitan regions (Supplementary Table 2).

**Figure 1.**
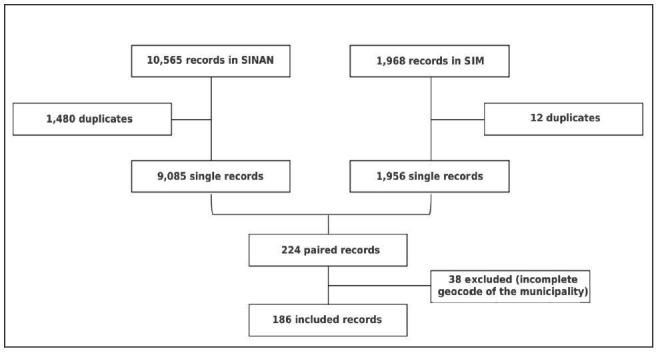
Linkage between databases of the Notifiable Diseases Information System and the Mortality Information System. Pernambuco, Brazil, 2007 to 2017.

SINAN: Notifiable Diseases Information System; SIM: Mortality Information System.

The number of individuals present in the linkage between the databases was 186 (Supplementary Table 3), corresponding to 2.05% of SINAN records and 9.51% of SIM records (Figure 1).

These cases are distributed in 55 municipalities in the state, 49 of which are considered endemic and 6 non-endemic territories. Regarding the municipality of origin, in the 186 records, 16.67% (31 individuals) are from Jaboatão dos Guararapes and 22 (11.83%), from Recife (Supplementary Table 3).

**Figure 2.**
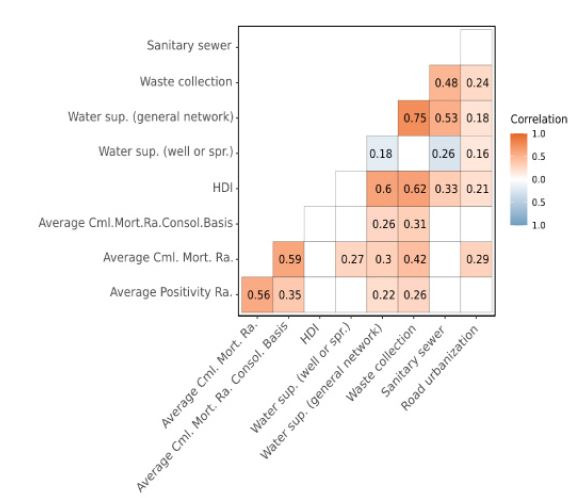
Pearson’s correlation matrix for the association between the average positivity rate of schistosomiasis and the average cumulative mortality rate with sociodemographic/sanitation variables. Pernambuco, Brazil, 2007 to 2017.

In Figure 2 we show the Pearson’s correlation matrix, in which the average positivity rate of schistosomiasis had a moderate positive correlation with the average cumulative mortality rate (r=0.56); and weak positive correlation with the average cumulative mortality rate of the linkage between databases (r=0.35). Among the sociodemographic and sanitation variables, the average positivity rate had a weak positive correlation with water supply by general network (r=0.22) and waste collection (r=0.26). For correlations between the average cumulative mortality rate and the sociodemographic and sanitation variables, a weak positive correlation was verified with the water supply by well or spring (r=0.27) and with the water supply by general network (r=0.30); a moderate positive correlation with waste collection (r=0.42); and a weak positive correlation with road urbanization (r=0.29). Finally, the average cumulative mortality rate of the linkage between databases had a weak positive correlation with the water supply by general network (r=0.26) and with waste collection (r=0.31).

Ra.: Rate; Sup.: Supply; Spr.: Spring; Mort.: Mortality; Cml.: Cumulative; Consol.: Consolidated.

Considering the study period, the year with the highest occurrence of severe forms was 2007, with 3,153 recorded notifications (34.71%). The lowest occurrence was in 2016, with 218 (2.40%). As for deaths, the year with the highest occurrence was 2010, with 205 (10.48%), and the lowest occurrence dates from 2014, with 155 (7.92%). For the linkage between databases, the highest records were in 2015, with 33 (17.74%), and the lowest in 2008, with six deaths (3.23%).

**Figure 3.**
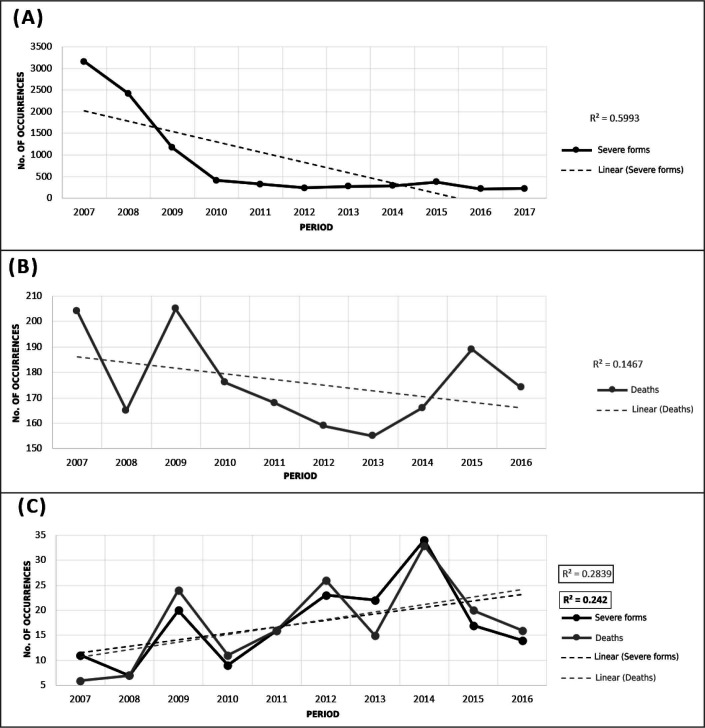
Linear trend graphs with regression line for severe forms of schistosomiasis (A), deaths from schistosomiasis (B), and *linkage* between databases (C). Pernambuco, Brazil, 2007 to 2017.

In Figure 3A, we observe a decline in severe forms of schistosomiasis until mid-2010, when its occurrence reaches a plateau that extends to the last period of the study. The regression coefficient obtained was 0.5993, representing a considered variability of severe forms in relation to the average.

In Figure 3B, we observe fluctuations in the behavior of deaths, accumulated in 2007 and 2008, decreased in 2009, and increased in 2010. It remains decreasing until 2014, increasing again in 2015 and 2016, and decreasing in 2017. The obtained regression coefficient was 0.2255, demonstrating low variability of deaths in relation to their average.

In Figure 3C, we verify an oscillation of the linkage between databases, alternating between decreases and increases in occurrence throughout the period. The regression line in this last graph obtained a coefficient of 0.2546 for severe forms; and of 0.3035 for deaths, indicating a low variability in relation to the averages.

## DISCUSSION

The results of our study presented the situation of Pernambuco regarding the records of severe forms and deaths from schistosomiasis as well as the main correlated sociodemographic factors in its transmission and occurrence in a time series.

By the technique of probabilistic linkage between databases, we could observe that, from 2007 to 2017, the majority of deaths from schistosomiasis were not notified in SINAN. This result corroborates the study by Oliveira et al.^27^, who identified in the city of Recife (state of Pernambuco) 238 deaths from schistosomiasis, in the period from 2007 to 2013; and of these, only 23 were paired in SINAN. Other studies that addressed infectious diseases^28,29^ point to the failure to integrate deaths recorded in SIM with SINAN, showing an invisibility of the occurrence of deaths due to certain diseases, with consequent damage to the planning of control actions and health management. To increase the sensitivity of the health information system in the identification of individuals infected with schistosomiasis, SINAN and SIM must be used in an integrated way, reducing underreporting and qualifying causes of death^27^, because, even though the SIM is considered the most reliable data source in the country, continuous stimuli are necessary to improve the quality of completing records, reducing fields with ignored, blank, and inconsistent data^18,30^.

In this study, the average positivity rate of schistosomiasis was moderately positively correlated with the average cumulative mortality rate, in such a way that places with positivity for the disease consequently result in deaths. This information stresses the weaknesses in timely diagnosis and treatment, which favors the maintenance of the transmission cycle of the disease and its evolution to severe clinical forms. It is worth highlighting the weak and moderate correlations between dependent and independent variables, which reinforces the understanding that schistosomiasis is a neglected disease with strong social, environmental, and poverty-related determination^6,8,31^.

Studies highlight the association of sanitation and environmental variables, such as HDI, basic sanitation, and waste collection, with the incidence of the disease and maintenance of the transmissibility of schistosomiasis^32,33^. In our study, despite the lack of associations and moderate and weak associations between positivity and mortality from *S. mansoni* with the factors (water supply, waste collection, HDI, road urbanization, and sanitary sewer), it is known that effective control of schistosomiasis depends on the solution to sanitation and socioeconomic problems^34^.

The number of notifications in SINAN was higher in 2007 (3,153), with a decrease over the period. This result may be related to underreporting, a reality found in other secondary database studies with this HIS^27-29^. This finding can also be associated with advances in health services over the years, with tests performed and treatment of positive cases, especially in endemic territories^35,36^.

The year that presented the highest occurrence of deaths in SIM was 2010 (205); however, when analyzing the temporal trend, we observed a low annual variability, according to the study by Silva et al.^37^, who analyzed schistosomiasis mortality in the state of Pernambuco from 2011 to 2019 and found that the mortality rate did not vary in the period. Studies show that, even with the epidemiological and control actions developed by the PCE in endemic areas, the disease still persists in the state, evolving to severe forms and deaths^13,27,36-39^.

This reality can be attributed to the failure of the articulation between the actions of the PCE and health surveillance and primary health care in the health units of the municipalities, in addition to the focus of actions often centered on mass medicalization, which temporarily combats the indicators of the disease. In order to avoid reinfection and achieve a decrease in the number of deaths and severe clinical forms, its transmission must be controlled, with interruption of the evolutionary cycle of the parasite. This may be possible through government actions, such as proper sanitation installation, with water and sewage in homes, changes in the environment, health education, vector control, in addition to timely diagnosis and treatment of the infected people^9,40-42^.

The use of secondary data is pointed out as a limiting factor in studies such as ours, considering the inclination to biases such as duplicates, incorrect and/or incomplete typing of the data, and possible losses in the process between data collection and its inclusion in the HIS.

The vast majority of deaths recorded in SIM did not have notification in SINAN, which denotes fragility in the integration between the HIS, mainly pointing to underreporting and the need for qualification in completing the information and defining the causes of death. Thus, we emphasize the need for these systems to be used in an associated way to increase sensitivity in the identification of severe cases and deaths from schistosomiasis and that, therefore, more effective actions are developed aimed at controlling the disease.

The results demonstrate that the precariousness in water supply, waste collection, and sanitary sewer are potential maintainers of mortality from the disease in the state. Therefore, the eradication of schistosomiasis as a public health issue will only be possible with investments in effective intersectoral public policies mainly aimed at health education and at reducing and improving social inequalities and living conditions of the population, in which individuals can be empowered according to their realities and modify the status quo in which inequities in public health are socially multiplied, either by changes in their habits or by adhering to the activities proposed by health programs.

Our findings showed a temporal trend of decrease in the records of severe cases of schistosomiasis, indicating improvements in the developed actions, but it may also suggest underreported cases that, with the oscillation of the occurrence of deaths observed during the study period, stress the need for greater investments in disease control and information quality.
