# Supplementary material for: Factors associated with severe forms and deaths from schistosomiasis and application of probabilistic linkage in databases, state of Pernambuco, Brazil, 2007–2017
Source: Rev Bras Epidemiol. 2023 Jan 9;26:e230003. doi: 10.1590/1980-549720230003.2 (PMC9838233; doi:10.1590/1980-549720230003.2)
Supplement: Supplementary file 1 [file 1980-5497-rbepid-26-e230003-s1.pdf]

Tabela 1 - Número de casos e taxa média de positividade da esquistossomose por município e ano de ocorrência. Pernambuco, 2007 a 2017.

Município	Número de Casos (N)	Número de Casos (%)	Taxa Média de Positividade
Abreu e Lima	37	0,41	3,45
Agrestina	48	0,53	18,5
Água Preta	80	0,88	21,07
Águas Belas	7	0,08	1,53
Alagoinha	1	0,01	0,65
Aliança	46	0,51	10,92
Altinho	28	0,31	11,12
Amaraji	163	1,79	65,76
Angelim	9	0,10	7,72
Araçoiaba	8	0,09	3,82
Araripina	1	0,01	0,11
Arcoverde	3	0,03	0,38
Barra de Guabiraba	106	1,17	72,25
Barreiros	350	3,85	75,94
Belém de Maria	67	0,74	51,97
Belo Jardim	1	0,01	0,12
Bezerros	29	0,32	4,38
Bom Conselho	17	0,19	3,29
Bom Jardim	42	0,46	9,84
Bonito	72	0,79	17,04
Brejão	2	0,02	2,01
Brejo da Madre de Deus	7	0,08	1,35
Buenos Aires	51	0,56	35,94
Buíque	2	0,02	0,33
Cabo de Santo Agostinho	96	1,06	4,51
Cachoeirinha	8	0,09	3,73
Caetés	1	0,01	0,33
Calçado	1	0,01	0,8
Camaragibe	49	0,54	2,97
Camocim de São Félix	17	0,19	8,69
Camutanga	27	0,30	29,26
Canhotinho	6	0,07	2,18
Capoeiras	1	0,01	0,45
Carnaíba	1	0,01	0,48
Carpina	162	1,78	18,84
Caruaru	139	1,53	3,82
Catende	125	1,38	28,66
Chã de Alegria	31	0,34	21,88
Chã Grande	17	0,19	7,41

Condado	12	0,13	4,33
Correntes	9	0,10	4,61
Cortês	182	2,00	130,24
Cupira	79	0,87	30,39
Escada	807	8,88	111,42
Feira Nova	7	0,08	2,99
Ferreiros	6	0,07	4,62
Frei Miguelinho	2	0,02	1,23
Gameleira	5	0,06	1,56
Garanhuns	3	0,03	0,2
Glória do Goitá	167	1,84	50,78
Goiana	76	0,84	8,9
Gravatá	498	5,48	56,91
Iati	52	0,57	25,04
Ibimirim	2	0,02	0,65
Igarassu	37	0,41	3,14
Iguaracy	1	0,01	0,75
Ilha de Itamaracá	4	0,04	1,56
Ipojuca	9	0,10	0,96
Itacuruba	1	0,01	1,99
Itaíba	2	0,02	0,68
Itambé	276	3,04	69,17
Itapetim	1	0,01	0,65
Itapissuma	22	0,24	8,05
Itaquitinga	424	4,67	232,38
Jaboatão dos Guararapes	641	7,06	8,71
Jaqueira	56	0,62	43,35
Jataúba	2	0,02	1,1
João Alfredo	2	0,02	0,57
Joaquim Nabuco	45	0,50	25,39
Jupi	1	0,01	0,64
Jurema	17	0,19	10,31
Lagoa de Itaenga	2	0,02	0,86
Lagoa do Carro	36	0,40	19,53
Lagoa do Ouro	5	0,06	3,61
Lagoa dos Gatos	3	0,03	1,7
Lajedo	15	0,17	3,58
Limoeiro	14	0,15	2,25
Macaparana	119	1,31	43,83
Machados	95	1,05	59,96
Manari	2	0,02	0,95
Maraial	4	0,04	2,97
Moreno	9	0,10	1,38

Nazaré da Mata	233	2,56	66,76
Olinda	45	0,50	1,05
Orobó	42	0,46	16,24
Ouricuri	2	0,02	0,27
Palmares	346	3,81	51,2
Palmeirina	10	0,11	10,86
Panelas	103	1,13	35,2
Paranatama	5	0,06	4,01
Passira	9	0,10	2,8
Paudalho	83	0,91	14,12
Paulista	30	0,33	0,87
Pedra	1	0,01	0,42
Pesqueira	1	0,01	0,14
Petrolândia	4	0,04	1,07
Petrolina	2	0,02	0,06
Poção	1	0,01	0,81
Pombos	147	1,62	50,09
Primavera	77	0,85	50
Quipapá	35	0,39	12,71
Recife	605	6,66	3,46
Riacho das Almas	25	0,28	11,47
Ribeirão	11	0,12	2,18
Rio Formoso	4	0,04	1,59
Sairé	35	0,39	28,54
Salgadinho	15	0,17	13,86
Saloá	28	0,31	16,21
Sanharó	2	0,02	0,78
Santa Cruz do Capibaribe	139	1,53	13,48
Santa Maria da Boa Vista	3	0,03	0,67
Santa Maria do Cambucá	12	0,13	8,08
São Benedito do Sul	1	0,01	0,62
São Bento do Uma	6	0,07	0,98
São Caitano	12	0,13	3
São João	33	0,36	13,62
São Joaquim do Monte	107	1,18	46,2
São José da Coroa Grande	27	0,30	12,78
São José do Egito	1	0,01	0,28
São Lourenço da Mata	47	0,52	3,99
São Vicente Ferrer	48	0,53	24,88
Serra Talhada	2	0,02	0,22
Serrita	1	0,01	0,48
Sertânia	1	0,01	0,26
Sirinhaém	3	0,03	0,65

Surubim	11	0,12	1,64
Tacaimbó	2	0,02	1,4
Tamandaré	52	0,57	21,79
Taquaritinga do Norte	11	0,12	3,81
Terezinha	6	0,07	7,84
Terra Nova	1	0,01	0,93
Timbaúba	356	3,92	59,26
Toritama	153	1,68	36,25
Tracunhaém	54	0,59	36,5
Trindade	1	0,01	0,33
Vertente do Lério	2	0,02	2,29
Vertentes	23	0,25	10,94
Vicência	122	1,34	34,97
Vitória de Santo Antão	334	3,68	22,87
Xexéu	3	0,03	1,88
**Total**	**9.085**	**100,00**	**9,05**

* Taxa média de positividade por 100.000 habitantes.

Tabela 2 - Número de óbitos e taxa média de mortalidade acumulada da esquistossomose por município e ano de ocorrência. Pernambuco, 2007 a 2017.

Município	Número de Óbitos (N)	Número de Óbitos (%)	Taxa Média de Mortalidade Acumulada
Abreu e Lima	34	1,74	3,17
Agrestina	10	0,51	3,85
Água Preta	18	0,92	4,74
Águas Belas	4	0,20	0,87
Aliança	27	1,38	6,41
Altinho	6	0,31	2,38
Amaraji	6	0,31	2,42
Angelim	1	0,05	0,86
Araçoiaba	17	0,87	8,13
Arcoverde	2	0,10	0,25
Barra de Guabiraba	6	0,31	4,09
Barreiros	24	1,23	5,21
Belém de Maria	8	0,41	6,21
Belo Jardim	6	0,31	0,73
Bezerros	19	0,97	2,87
Bom Conselho	20	1,02	3,87
Bom Jardim	7	0,36	1,64
Bonito	6	0,31	1,42
Brejão	1	0,05	1,01
Brejo da Madre de Deus	7	0,36	1,35
Buenos Aires	8	0,41	5,64
Buíque	1	0,05	0,17
Cabo de Santo Agostinho	56	2,86	2,63
Cachoeirinha	1	0,05	0,47
Caetés	1	0,05	0,33
Calçado	2	0,10	1,61
Camaragibe	51	2,61	3,09
Camocim de São Félix	3	0,15	1,53
Camutanga	1	0,05	1,08
Canhotinho	9	0,46	3,27
Capoeiras	1	0,05	0,45
Carpina	38	1,94	4,42
Caruaru	42	2,15	1,15
Catende	27	1,38	6,19
Chã de Alegria	4	0,20	2,82
Chã Grande	7	0,36	3,05
Condado	9	0,46	3,25
Correntes	3	0,15	1,54

Cortês	4	0,20	2,86
Cumaru	4	0,20	2,26
Cupira	6	0,31	2,31
Escada	42	2,15	5,80
Feira Nova	4	0,20	1,71
Ferreiros	2	0,10	1,54
Floresta	1	0,05	0,30
Frei Miguelinho	4	0,20	2,45
Gameleira	22	1,12	6,87
Garanhuns	14	0,72	0,95
Glória do Goitá	14	0,72	4,26
Goiana	30	1,53	3,51
Gravatá	19	0,97	2,17
Igarassu	21	1,07	1,78
Ilha de Itamaracá	4	0,20	1,56
Ipojuca	25	1,28	2,66
Itaíba	1	0,05	0,34
Itambé	24	1,23	6,02
Itapissuma	2	0,10	0,73
Itaquitinga	14	0,72	7,67
Jaboatão dos Guararapes	146	7,46	1,98
Jaqueira	5	0,26	3,87
João Alfredo	5	0,26	1,42
Joaquim Nabuco	9	0,46	5,08
Jurema	1	0,05	0,61
Lagoa de Itaenga	14	0,72	6,00
Lagoa do Carro	8	0,41	4,34
Lagoa do Ouro	2	0,10	1,45
Lagoa dos Gatos	1	0,05	0,57
Lajedo	2	0,10	0,48
Limoeiro	32	1,64	5,14
Macaparana	12	0,61	4,42
Machados	10	0,51	6,31
Maraial	3	0,15	2,23
Moreno	25	1,28	3,85
Nazaré da Mata	29	1,48	8,31
Olinda	73	3,73	1,71
Orobó	7	0,36	2,71
Palmares	43	2,20	6,36
Palmeirina	1	0,05	1,09
Panelas	5	0,26	1,71
Passira	8	0,41	2,49
Paudalho	22	1,12	3,74

Paulista	64	3,27	1,86
Pedra	1	0,05	0,42
Pesqueira	2	0,10	0,28
Petrolândia	1	0,05	0,27
Petrolina	1	0,05	0,03
Pombos	4	0,20	1,36
Primavera	3	0,15	1,95
Quipapá	3	0,15	1,09
Recife	364	18,61	2,08
Riacho das Almas	1	0,05	0,46
Ribeirão	20	1,02	3,96
Rio Formoso	9	0,46	3,58
Sairé	1	0,05	0,82
Salgueiro	2	0,10	0,31
Saloá	2	0,10	1,16
Sanharó	1	0,05	0,39
Santa Cruz do Capibaribe	5	0,26	0,48
Santa Maria do Cambucá	1	0,05	0,67
São Benedito do Sul	5	0,26	3,11
São Bento do Uma	3	0,15	0,49
São Caitano	2	0,10	0,50
São João	4	0,20	1,65
São Joaquim do Monte	5	0,26	2,16
São José da Coroa Grande	6	0,31	2,84
São Lourenço da Mata	45	2,30	3,82
São Vicente Ferrer	4	0,20	2,07
Serra Talhada	3	0,15	0,33
Sirinhaém	9	0,46	1,94
Solidão	1	0,05	1,54
Surubim	12	0,61	1,79
Tacaimbó	1	0,05	0,70
Tamandaré	3	0,15	1,26
Taquaritinga do Norte	2	0,10	0,69
Timbaúba	43	2,20	7,16
Toritama	8	0,41	1,90
Tracunhaém	9	0,46	6,08
Triunfo	1	0,05	0,59
Vertente do Lério	2	0,10	2,29
Vertentes	3	0,15	1,43
Vicência	21	1,07	6,02
Vitória de Santo Antão	55	2,81	3,77

Xexéu	11	0,56	6,90
**Total**	**1.956**	**100,00**	**1,95**

* Taxa média de mortalidade acumulada por 100.000 habitantes.

Tabela Suplementar 3 - Número de óbitos e taxa média de mortalidade acumulada da esquistossomose após *linkage* entre as bases de dados por município de procedência. Pernambuco, 2007 a 2017.

**Município**	**Número de óbitos (N)**	**Número de óbitos (%)**	**Taxa de Mortalidade Média Acumulada**
Abreu e Lima	5	2,69	0,47
Agrestina	2	1,08	0,77
Água Preta	2	1,08	0,53
Aliança	3	1,61	0,71
Altinho	1	0,54	0,40
Angelim	1	0,54	0,86
Araçoiaba	1	0,54	0,48
Barreiros	1	0,54	0,22
Bezerros	2	1,08	0,30
Bom Jardim	1	0,54	0,23
Brejo da Madre de Deus	1	0,54	0,19
Cabo de Santo Agostinho	8	4,30	0,38
Cachoeirinha	1	0,54	0,47
Camaragibe	5	2,69	0,30
Carpina	2	1,08	0,23
Caruaru	2	1,08	0,05
Catende	1	0,54	0,23
Chã Grande	2	1,08	0,87
Correntes	1	0,54	0,51
Escada	5	2,69	0,69
Feira Nova	1	0,54	0,43
Glória do Goitá	2	1,08	0,61
Goiana	1	0,54	0,12
Igarassu	3	1,61	0,25
Ilha de Itamaracá	1	0,54	0,39
Ipojuca	4	2,15	0,42
Itambé	3	1,61	0,75
Itaquitinga	2	1,08	1,10
Jaboatão dos Guararapes	31	16,67	0,42
Lagoa de Itaenga	2	1,08	0,86
Lagoa do Carro	3	1,61	1,63
Lagoa do Ouro	1	0,54	0,72
Limoeiro	6	3,23	0,96
Macaparana	1	0,54	0,37
Machados	1	0,54	0,63
Moreno	3	1,61	0,46
Nazaré da Mata	5	2,69	1,43
Olinda	4	2,15	0,09
Palmeirina	1	0,54	1,09
Passira	1	0,54	0,31
Paudalho	1	0,54	0,17
Paulista	5	2,69	0,15
Pedra	1	0,54	0,42
Recife	22	11,83	0,13
Rio Formoso	1	0,54	0,40

Santa Cruz do Capibaribe	2	1,08	0,19
São Caitano	1	0,54	0,25
São Joaquim do Monte	1	0,54	0,43
São Lourenço da Mata	7	3,76	0,59
Surubim	2	1,08	0,30
Timbaúba	4	2,15	0,67
Toritama	4	2,15	0,95
Vicência	4	2,15	1,15
Vitória de Santo Antão	7	3,76	0,48
Xexéu			

* Taxa média de mortalidade acumulada por 100.000 habitantes.
